# Improving Resident Doctor Confidence in Managing Emergency Physical Health Needs of Psychiatric Inpatients Out of Hours

**DOI:** 10.1192/bjo.2025.10442

**Published:** 2025-06-20

**Authors:** Sushmita Sidhu, Tania Rahman, Rohan Krishnan, Ali Iqbal, Hester Mannion

**Affiliations:** 1 North East London NHS Foundation Trust, London, United Kingdom; 2 East London NHS Foundation Trust, London, United Kingdom

## Abstract

**Aims:** Resident doctors in inpatient psychiatric settings manage urgent or emergency physical healthcare out of hours with limited, remote guidance and support, creating a potentially stressful and isolative work environment. The primary outcome of this Quality Improvement (QI) project is to address this problem by enhancing the confidence of resident doctors working out of hours in psychiatric inpatient units. Secondary goals include improving morale, fostering connections with acute medical teams, and delivering more timely and targeted physical health care for psychiatric inpatients with urgent physical health needs.

**Methods:** The team used a structured QI approach, including constructing a survey-informed driver diagram and PDSA cycles. Data collection involved surveys of resident doctors at the beginning and end of a 6-month rotation across two inpatient psychiatric units within East London NHS Foundation Trust. Surveys aimed to identify confidence gaps, barriers, and existing challenges in managing physical health emergencies out of hours. Interventions implemented were designed to address these gaps and included:

Awareness and education: Promoting resources such as the BMJ Best Practice app for quick guidance. Emergency grab bag familiarisation sessions.

Integration of physical health updates: Including relevant physical health specialty sessions in the academic programme for resident doctors. Also enhancing connection with locality acute medical teams.

Working with locality physical health lead nurse: To involve resident doctors with ward-based simulation sessions already operating.

**Results:** Quantitative and qualitative results highlight changes in confidence levels, resource utilisation, and satisfaction among junior doctors, with 19 respondents in the initial survey and 13 in the final survey.

The post-survey showed improved confidence in managing physical health on call.

Post-intervention, respondents found the most value in the updated academic programme (76.9%), and strengthened links with acute teams (76.9%), followed by emergency grab bag familiarisation sessions (61.5%), the BMJ app (61.5%), and ward-based simulation sessions (46.2%).

**Conclusion:** Improved awareness of resources, physical health updates, and closer working relationships with acute medics and nurses can improve resident doctors’ confidence in managing physical health emergencies in inpatient settings.

This initiative has the potential to improve provider satisfaction and patient outcomes. The next steps include expanding interventions to the final part of the borough in the next trainee changeover and establishing a new 6-month Physical Health Representative resident doctor role to sustain momentum through continuous improvement and support the development of trainee leadership skills in this vital improvement area.

